# Effects of four extenders on the quality of frozen semen in Arabian stallions

**DOI:** 10.14202/vetworld.2019.34-40

**Published:** 2019-01-07

**Authors:** Mohaammed Saad Alamaary, Abd Wahid Haron, Mohamed Ali, Mark Wen Han Hiew, Lawan Adamu, Innocent Damudu Peter

**Affiliations:** 1 Department of Veterinary Clinical Studies, Faculty of Veterinary Medicine, Universiti Putra Malaysia, UPM, 43400 Serdang, Selangor Darul Ehsan, Malaysia; 2 King Abdulaziz Arabian Horses Center, Ministry of Agriculture, Riyadh, Saudi Arabia; 3 Department of Animal Production and Breeding, Faculty of Agriculture and Veterinary Medicine, AL Qassim University, AL Qassim State, Saudi Arabia; 4 Faculty of Veterinary Medicine, University of Maiduguri, Maiduguri, Nigeria

**Keywords:** Arabian stallion, frozen semen, semen, extenders

## Abstract

**Aim:**

Different types of extenders have a variety of components which show the tolerance effect on sperm protection during freezing procedures. In the present study, we have examined the impact of the extenders HF-20 and Tris, which were locally manufactured, and they are competing with commercial extenders INRA Freeze^®^ (IMV Technologies, France) and EquiPlus Freeze^®^ (Minitube, Germany) on the quality of horses frozen semen.

**Materials and Methods:**

A total of 15 ejaculates from three healthy stallions were collected and cryopreserved in the same environment. Each semen sample collected was divided into four equal parts and processed. All samples were analyzed before and after freezing for motility, viability, plasma membrane integrity, and morphology. Furthermore, twenty mares were inseminated using post-thawed semen.

**Results:**

There were no differences observed among all extenders in all the parameters before freezing. Sperm cryopreserved using HF-20 showed better motility, viability, and plasma membrane integrity than Tris extender. The Tris extender showed the most inferior quality of post-thawed semen between all the extenders. HF-20, INRA Freeze^®^, and EquiPlus Freeze^®^ extenders revealed the same capacity of semen preservation *in vitro* and *in vivo*.

**Conclusion:**

HF-20 extender has the same quality as INRA Freeze^®^ and EquiPlus Freeze^®^ that can be considered as one of the best extenders for the semen cryopreservation in horses. In contrast, Tris extender needs some degree of improvement.

## Introduction

Artificial insemination (AI) has quickened the genetic improvement due to the comfort involved in transporting the semen from the insemination center to the place where the mare is inseminated which offers a broader choice to breeders outside the country. Therefore, the use of AI becomes the basic technique in the modern horse industry. Furthermore, the number of mares that can be impregnated by one stallion during the breeding season will increase due to the possibility of dividing the ejaculate into several insemination doses. Frozen semen has more benefit than fresh or chilled semen; therefore, this makes it popular in the horse industry. The use of frozen semen could preserve horse’s genetics for unbounded time and enables to maintain the semen for the future in any event such as death. Furthermore, cryopreserved semen is a basic approach in the modern assisted reproductive technology such as *in vitro* fertilization and intracytoplasmic sperm injection.

However, most of the Arabian mares in the Kingdom of Saudi Arabia were bred naturally [1]. Unfortunately, a considerable number of stallions have poor post-thawed semen quality and fertility [2]. Besides, horses showed high variations between stallions and ejaculates on sperm parameters and pregnancy rate of frozen semen [3]. Semen pH is one of the essential factors that affect the semen quality due to its effect on sperm motility and metabolism [4]. Lactic acid is produced by the glycolytic metabolism of the sperm that leads to diminished pH in the semen sample [5].

Therefore, more consideration is required for semen extender to improve the frozen semen quality for horses. Different types of buffers could be supplemented to the semen extender to balance the pH during semen preservation. The variation of buffer capacity may have an effect on the sperm biological system and enzymes [6]. As a result of the high molecular weight of sugars such as glucose, fructose, and raffinose, therefore, these buffers will not be able to penetrate the sperm membrane. Thus, sugars decrease the concentration of cryoprotectant required for cryopreservation. Besides the ability of sugars to dissolve the cryoprotectant after cryopreservation, it also reduces the osmotic shock [7,8].

Therefore, the current study aims to determine the best extender which contained different types of buffers, Tris buffer in Tris-based extender and raffinose, glucose, and lactose buffers in HF-20 extender, that were locally manufactured compared with commercial extenders like INRA Freeze and EquiPlus.

## Materials and Methods

### Ethical approval

This study was exempted approval from the Institution Animal Ethics because the semen collection using artificial vagina does not affect the normal physiology of the animal.

### Animals

Three healthy stallions aged 4-10 years and twenty mares aged 3-12 years were selected after the breeding soundness examination. All mares were with a good general physical condition without any history of reproductive failures or disturbances. The external and internal genitalia were examined using the rectal palpation and ultrasonography to avoid any disturbance in the reproductive tract that might affect mare’s fertility. All animals have housed individually in Horses Research Unit at the farm of Qassim University and fed pellets supplemented with berseem (*Trifolium alexandrinum*). Epididymal sperm reserves were decreased by the semen collection daily in all stallions for 3 days. After that, a rhythm semen collection twice a week per stallion was performed using artificial vagina (AV). Fifteen ejaculates were collected in this experiment.

### Semen collection and processing

Missouri model AV was used for the semen collection, in this trial. The gel was removed immediately from the semen sample after semen collection using sterile gauze and then transferred to a water bath at 37°C. The semen volume was measured in a graduated cylinder. After that, the ejaculate was evaluated for general and progressive motility and sperm concentration. Sperm concentration and motility were determined using CASA system (ISAS^®^ program, Prosser R+D, Paterna, Valencia, Spain). Sample with minimum concentration of 200×10^6^/ml and motility >50% was selected for the study.

Filtered semen of each ejaculate was diluted (1:1) with centrifugation medium containing 6.0 g glucose, 0.37 g ethylenediaminetetraacetic acid, 0.37 g sodium citrate, 0.12 g sodium bicarbonate, 100,000 IU penicillin, and 0.08 g streptomycin in 100 mL distilled water. The mixture was centrifuged at 800 g for 10 min to remove seminal plasma, divided into four aliquots, and resuspended with FH-20, Tris, INRA Freeze, or EquiPlus extenders (Table-1). The final semen concentration after dilution was 200×10^6^ sperm/ml. All the tubes were cooled to 4°C for 90 min before assessing for the motility, morphology, and sperm membrane integrity. The straws were filled with 0.5 ml of the cooled semen samples. Then, the straws were placed 9 cm above the level of the liquid nitrogen for 9 min before plunging into the liquid nitrogen and stored at −196°C.

**Table-1:** Semen extenders used on each group.

E1 – Tris	E2 - HF-20	E3 - EquiPlus	E4 - INRA
Tris 2.42 g	Glucose 5 g	Part A: 95 ml medium	INRA freeze
Citric acid 1.34 g	Lactose 0.3 g	Part B: 5 ml egg yolk-glycerol component	(France IMV Technologies)
Fructose 1 g	Raffinose 0.3 g	Minitube - Animal Reproduction Technologies (Germany)	
Streptomycin 0.08 g	Sodium citrate 0.15 g		
Glycerol 7 ml	Sodium phosphate 0.05 g		
Egg yolk 20 ml	Potassium sodium tartrate 0.05 g		
Deionized water (made up to 100 ml)	Egg yolk 10%		
	Penicillin 25.000 IU		
	Streptomycin 0.08 μg		
	Glycerol 3%		
	Deionized water (made up to 100 ml)		

### Extenders

Four types of extenders were used to resuspend the semen samples (Table-1).

### Frozen semen evaluation

Water bath at 38°C for 60 s was used to thaw frozen straws, and every straw was expelled in a small warm tube. After that, evaluation of the general and progressive motility using ISAS program was conducted first and then assessed for plasma membrane integrity, morphology defects, and viability.

### Assessment of sperm motility

The ISAS^®^ program (CASA system) was used to assess the motility pattern either after immediate dilution of the semen or post-thawed semen. A sample (2.7 µL) of each tube was put in a pre-warmed slide, and then, the semen motility was observed based on five digitalized images from different fields through a 10× negative-phase contrast objective and warm stage at 38°C. Motility pattern was observed according to the total motile sperms (%), rapid progressively motile (RPS %), curvilinear speed (VCL µm/s), rectilinear speed (VSL µm/s), average value (VAP µm/s), linearity index (LIN %), and the straightness index (STR %). The sperm motility parameters that were involved were the rapid motile sperm (RMS %), medium motile sperm (MMS %), and slow motile sperm (SMS %). The sperm progressive motility matches with the swimming speed of 10-45, 45-90, and >90 µm/s, respectively. Sperm with a VAP value ≤10 µm/s and a swimming speed <10 µm/s was considered immotile sperm. At least 300 sperms were analyzed in each sample, and the images were read within 1 s.

### Plasma membrane integrity

Hypoosmotic swelling test (HOST) was used to assess the plasma membrane integrity of spermatozoa. A minimum of 100 sperms was analyzed for the presence or absence of a coiled tail using phase-contrast microscopy (400×). A mixture of a sucrose-based solution at 100 mOsmol and 20 µL of semen was incubated at 37°C for 50 min in a water bath [9].

### Vital test

Sperm viability was evaluated using acridine orange (AO) and propidium iodide (PI) staining kit (Halotech DNA S.L., Spain). First of all, semen was diluted to 10-15×10^6^ sperm/ml, and then, 10 µL of diluted semen was placed onto a clean slide. After that, 1.0 µL of AO and 1.0 µL of PI were mixed with the diluted semen; finally, the mixture was covered by coverslip and then evaluated on a fluorescence microscope. The living sperm retained the AO giving green fluorescence, while PI penetrated the damaged sperm causing red fluorescence. A total of 300 sperms were assessed per sample.

### Morphology

Sperm morphology was examined using eosin-nigrosin staining technique (RAL Diagnostics, Martillac, France). A drop of 15 µL of semen was mixed with 15 µL of eosin-nigrosin on a clean and warmed slide. The mixture was gently spread and evaluated randomly under oil immersion 1000× magnification. The spermatozoa morphological defects were classified according to normal and abnormal acrosome, abnormal head, abnormal midpiece, abnormal tail, head detached, proximal droplet, distal droplet, bent tail, and others [10].

### AI of mares

Five mares were inseminated with each extender from a total of twenty mares that were used in this experiment. Each mare was inseminated randomly from one of the four post-thawed semen extenders. Mares with a follicle ≥35 mm and uterine edema which were in estrus are inseminated. All mares were injected with 3000 IU of human chorionic gonadotropin intravenously to stimulate the ovulation, and then, the mares were monitored every 6 h starting at 18 h after the injection. Ovulation time was confirmed using ultrasound at 5 MHZ transrectal probe. Rectal palpation was performed to remove the fecal and determine the ovaries position before inserting the probe. Each ovary has been scanned, and the size of the follicles was registered. The mares were inseminated when the follicle identified as dominant disappeared. All insemination doses were deposited on the uterine body using a flexible pipette 65 cm (Minitube). The dose was 4 ml of 800×10^6^ total sperm (8 straws and each one contains 100×10^6^). One dose was deposited within a maximum of 6 h after ovulation. Pregnancy diagnosis was carried out at 15 days and 30 days post-insemination.

### Statistical analysis

One-way ANOVA was used to analyze the results of the general motility of the spermatozoa, progressive motility, VCL, VSL, linearity (LIN), straightness (STR), morphology abnormalities, viability, and plasma membrane integrity. Pregnancy rate data were analyzed using Chi-square test to describe the association between all the groups. The data obtained from the study were expressed statistically as mean±SEM, and the differences were identified at p<0.05. SPSS statistical software version 16.0 (IBM, USA) was used to analyze the data.

## Results

The data of the effects of different extenders after cooling for 90 min on sperm quality are shown in Tables-2 and 3. The result showed that no significant differences were observed in all the semen parameters (p>0.05), except on progressive motility which revealed a significant difference (p<0.05) on the Tris extender.

**Table-2:** Effect of different extenders on chilled stallion semen after 90 min on the motility pattern.

Parameters	HF - 20	INRA freeze	EquiPlus	TRIS
Motility (%)	65.90±17.80^a^	73.00±17.10^a^	68.75±14.75^a^	39.40±7.40^a^
PM (%)	30.25±0.05^a^	38.80±3.20^a^	37.85±2.15^a^	11.32±2.32^b^
VCL (μm/s)	94.87±0.07^b^	100.87±0.22^a^	92.45±1.80^c^	97.70±0.70^ab^
VSL (μm/s)	27.27±2.22^a^	31.02±0.87^a^	32.30±5.20^a^	30.65±0.55^a^
VAP (μm/s)	49.42±5.32^a^	53.12±1.97^a^	53.97±2.17^a^	50.15±0.25^a^
LIN (%)	29.62±1.62^a^	30.52±1.12^a^	34.80±6.35^a^	31.95±0.15^a^
STR (%)	56.62±2.37^a^	58.22±3.57^a^	59.22±7.12^a^	61.10±0.15^a^

All values are expressed as mean±SE, values with different superscripts^abc^ across rows indicate significant differences at P<0.05. PM=Progressive motility, VCL=Curvilinear speed, VSL=Rectilinear speed, VAP=Average value, LIN=Linearity index, STR=Straightness index

**Table-3:** Effect of different extenders on chilled stallion semen after 90 min on the plasma membrane integrity and morphology.

Parameters (%)	HF - 20	INRA freeze	EquiPlus	TRIS
Membrane integrity	53.40±3.40^a^	55.20±2.60^a^	61.05±0.25^a^	57.04±2.04^a^
Normal morphology	72.15±8.85^a^	67.98±10.61^a^	66.75±7.95^a^	70.94±8.84^a^
Major abnormalities	4.00±3.10^a^	2.00±1.20^a^	1.90±1.10^a^	2.05±0.05^a^
Minor abnormalities	23.70±5.69^a^	29.85±9.45^a^	27.20±4.70^a^	26.94±8.76^a^

All values are expressed as mean±SE, values with different superscripts^abc^ across rows indicate significant differences at P<0.05

In Table-4, the analysis of the post-thawing general and progressive motility using the ISAS^®^ program (CASA system) showed a significantly higher (p<0.05) general motility in samples cryopreserved using HF-20 compared with Tris extender. There were no significant differences (p>0.05) in the general motility between HF-20, INRA Freeze, and EquiPlus extenders, and no significant differences (p>0.05) exist between INRA Freeze, EquiPlus, and Tris extenders. For the progressive motility and VCL, the extenders HF-20, INRA Freeze, and EquiPlus were significantly higher (p<0.05) compared with Tris. INRA Freeze, EquiPlus, and HF-20 were significantly higher (p<0.05) in VSL compared with Tris, and INRA Freeze was higher significantly (p<0.05) compared with HF-20. There were no significant differences (p<0.05) existing between EquiPlus and HF-20. Tris extender was significantly lower (p<0.05) in VAP compared with HF-20, INRA Freeze, and EquiPlus. Furthermore, INRA Freeze and EquiPlus were significantly higher (p<0.05) in LIN and STR compared with HF-20 and Tris.

**Table-4:** Effect of different extenders on the post-thawed stallion semen and on the motility pattern.

Parameters	HF - 20	INRA Freeze	EquiPlus	TRIS
Motility (%)	46.92±10.71^a^	40.92±10.47^ab^	36.85±2.93^ab^	17.76±5.07^b^
PM (%)	25.02±2.85^a^	25.50±3.06^a^	18.87±4.07^a^	7.06±2.75^b^
VCL (μm/s)	106.66±3.74^a^	109.51±9.21^a^	98.83±2.34^a^	46.10±7.33^b^
VSL (μm/s)	32.60±1.31^b^	50.22±9.17^a^	48.48±3.72^ab^	13.00±0.72^C^
VAP (μm/s)	60.71±0.53^a^	71.06±8.72^a^	68.43±2.17^a^	25.10±1.70^b^
LIN (%)	31.98±1.71^b^	44.37±4.66^a^	49.15±3.17^a^	29.83±5.34^b^
STR (%)	54.08±2.08^b^	68.38±5.15^a^	70.23±3.34^a^	52.10±4.41^b^

All values are expressed as mean±SE, values with different superscripts^abc^ across rows indicate significant differences at P<0.05. PM=Progressive motility, VCL=Curvilinear speed, VSL=Rectilinear speed, VAP=Average Value, LIN=Linearity index STR=Straightness index

**Figure-1:**
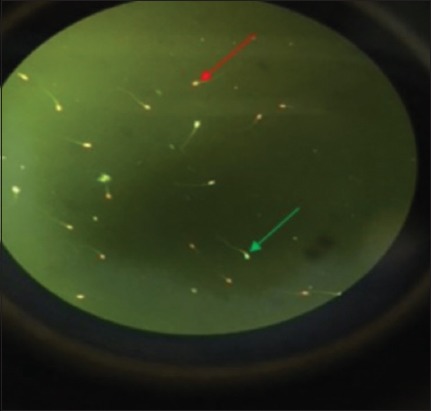
Live sperms appear green (green arrow) and dead appear in red (red arrow).

Table-5 shows the result of plasma membrane integrity, viability, and morphology. Plasma membrane integrity using HF-20 extender was significantly higher (p<0.05) compared with Tris, but there were no significant differences (p>0.05) existing between INRA Freeze and EquiPlus. Moreover, spermatozoa viability with the use of EquiPlus extender was greater compared with Tris but not significantly higher (p>0.05) compared with HF-20 and INRA Freeze (Figure-1). There were no significant differences (p>0.05) observed using HF-20, INRA Freeze, EquiPlus, and Tris for the normal and abnormal morphology.

**Table-5:** Effect of different extenders on the post-thawed stallion semen, the plasma membrane integrity, viability, and morphology.

Parameters (%)	HF - 20	INRA Freeze	EquiPlus	TRIS
Membrane integrity	44.49±10.82^a^	36.85±3.85^ab^	33.93±6.22^ab^	11.63±5.75^b^
Viability	32.38±14.27^ab^	32.65±9.05^ab^	48.05±5.85^a^	11.24±2.31^b^
Normal morphology	79.17±3.88^a^	79.55±2.03^a^	75.12±2.24^ab^	73.53±2.00^ab^
Major abnormalities	8.70±5.71^ab^	6.95±2.35^ab^	6.37±1.64^a^	8.70±2.03^a^
Minor abnormalities	11.57±1.88^a^	12.52±4.15^a^	18.35±1.53^a^	17.63±2.76^a^

All values are expressed as mean±SE, values with different superscripts^ab^ across rows indicate significant differences at P<0.05

The pregnancy rate of post-thawed semen using INRA Freeze, EquiPlus, HF-20, and Tris extenders was 40%, 60%, 40%, and 0%, respectively. Statistically, no differences were observed between INRA Freeze, EquiPlus, and HF-20.

## Discussion

The objective of the study was to determine the effect of Tris and HF-20 extenders on the frozen semen quality compared with INRA Freeze and EquiPlus extenders. In the present study, the procedure of semen collection, freezing, thawing, and insemination technique were controlled in all groups to eliminate the effect related to them. As the sample was from the same stallion and divided between the four groups and then processed similarly, the differences between the groups are only apparent due to the use of different extenders for each group.

In the present study, we used the sperm motility as the primary criterion to evaluate the stallion semen quality. This technique was similarly used by other authors [11-13] in their respective studies. Furthermore, in the present study, the variety of motility patterns such as percentages of motile sperm, progressively motile sperm, and sperm speed were evaluated using general motility, progressive motility, STR, and VCL as carried out by Bliss *et al.*, [14]. Therefore, to identify the suitable doses for AI in horses, progressive motility is an important parameter. Since the sperm needs to be transported to oviduct to meet and penetrate the oocyte, thus the percentage of sperms that have sufficient motility can indicate the semen fertility. The World Breeding Federation for Sport Horses [15] recommend a minimum 35% progressive motility and 750×10^6^ sperm/dose for post-thawed semen. This recommendation was supported by Barrier *et al.*, [16], who reported a high correlation between fertility and RPS. In the present study, there were no differences between all the groups before freezing. The movement pattern, sperm morphology, and sperm plasma membrane integrity were not affected after cooling for 90 min. This emphasizes the ability of lipoprotein in egg yolk in all the four different extenders to protect the sperm from the cold shock during freezing process. These findings are consistent with the results of previous studies [17].

Nevertheless, the extenders HF-20, INRA Freeze, and EquiPlus indicate the best post-thawed semen fertility correlated to great progressive motility, while the Tris extender showed the worst fertility in relation to progressive motility. This finding agrees with the result of Love, 2011, and Blanchard *et al.*, [18,19]. Furthermore, our result approves that addition of sugars on the extender can control the pH and provide the energy which enhances the sperm motility. Indicating fertility was associated highly with VSL when the sperm crosses a long distance in a short time, and average path velocity VAP of the sperm [20], which observed in HF-20, INRA Freeze, and EquiPlus extenders. The best results of post-thawed semen in HF-20 were detected for total motility (46.92), progressive motility (25.02), and VAP (60.71) on the same level with commercial extenders NIRA Freeze and EquiPlus.

Moreover, VSL of HF-20 was 32.60 which is higher than Tris and lower than INRA Freeze. Furthermore, VCL and LIN values are essential measures to identify the positive and negative motility pattern during sperm activity, whether progressive or circular movement. De Oliveira *et al*. [21] reported that a high value of VCL and low-value LIN are highly correlated with sperm fertility which was observed in HF-20, INRA Freeze, and EquiPlus extenders. In contrast, Tris buffer showed less protectant to the sperm capacity. The reason could be due to the high concentrations of sugars in HF-20 that provides energy to the extender which concurs with the previous study by Tuncer *et al*. [22].

The metabolism, osmolarity, and normal function of the sperm are related to sperm plasma membrane integrity. Therefore, the HOST is a critical test to determine the semen quality [23]. Since the integrity of sperm membrane is a necessary factor for sperm cryosurvival during semen cryopreservation, the HOST is valuable to analyze the fertility of horse semen [24]. According to the HOST result, in our study, HF-20 extender indicated the best sperm membrane integrity and was significantly higher compared with Tris. INRA Freeze, and EquiPlus extenders and did not show any significant differences with HF-20 nor Tris. Due to the osmotic buffer of sugars in the HF-20 extender, the osmotic shock induced by diluting the cryoprotectant after cryopreservation was the lowest in semen cryopreserved using HF-20. Our findings are in agreement with that of the study conducted by Garde *et al*. [25].

The thermal shock that occurs during cryopreservation in spermatozoa because of the modifications of sperm plasma membrane can be described using viability test. The type of substance such as sugars and cryoprotectant that were used in extender affects profoundly on the sperm motility [17]. These data describe the terrible effect of Tris extender during cryopreservation on the sperm viability that indicated the high sperm membrane damage. The reason for this disparity might be attributed to the toxic effects of Tris buffer due to its ability to penetrate the sperm membrane and change the intracellular metabolism.

The fertility of stallion semen associates extremely with sperm morphology [26]. Furthermore, analysis of the semen quality requires an accurate morphological assessment of the defects of individual sperm because some stallions may own high abnormal morphology with high semen motility [27]. In the present study, no differences were observed between all groups on morphological parameters. That emphasizes the performance of all extenders used to preserve the sperm morphology during semen cryopreservation. Which, agree with the reports of [28] who report no significant difference in sperm morphology between different extenders. The results of pregnancy rate support the *in vitro* parameters which were nil with Tris extender. The reason could be due to the poor sperm motility parameters on Tris extender comparing with the other extenders. In addition, this poor result of Tris extender might due to the small number of mares that were used for AI in this experiment. In contrast, the use of HF-20 extender resulted in 40% in pregnancy rate that is similar to the observation in INRA Freeze and EquiPlus. This result is within the range of fertility reported by others [29].

Tris-hydroxymethyl aminomethane has successfully been used on semen cryopreservation in a bull, ram, boar [30], and camel [31]. In the previous studies, on horses, Tris extender showed poor post-thawed semen quality of motility, viability, and sperm membrane integrity in horses [32]. Our findings confirmed the inadequate protection of Tris extender on horse semen. Tris extender is shown to have the lowest potential in preserving sperm motility, viability, and sperm membrane integrity than the other earlier mentioned extenders.

The ability of HF-20 extender to protect the semen during cryopreservation in the current study could have been due to the high sugar content in HF-20 extender. Because of the capability of sugars to dissolve the cryoprotectant that increases its ability to penetrate the sperm membrane. Besides providing the energy to sperm in the semen extenders. Semen samples cryopreserved with HF-20 show higher sperm membrane integrity and sperm motility patterns as compared to other extenders in this study. These results are in agreement with Tejpal *et al*. [33] and support by Bucak *et al*. [34] findings who reported a high protective effect of raffinose during semen preservation in ram and Naing *et al*. [35] who found definite improvement of frozen semen supplemented with sugars.

In the current study, we found tolerance performance among the extenders in motility, viability, sperm membrane integrity, and pregnancy rate in agreement with Loomis and Graham [36] who reported the effect of different types and concentration of buffers, sugars, salts, egg-yolk, and milk that used in extenders on the quality of post-thawed semen in horses. In goats, Akeel *et al*. [37] reported different post-thawed semen quality using different buffers. Furthermore, there is essential variation among species in sperm membrane composition and its permeability to a different type of extender that effect of sperm ability to survive during freezing procedure.

## Conclusion

Semen cryopreserved with HF-20 gave perfect result compared with commercial extenders (INRA Freeze and EquiPlus). Although HF-20 showed the highest quality of motility, there were no significant differences between HF-20, INRA Freeze, and EquiPlus. On the other hand, the use of Tris extender in semen cryopreservation in horses requires more improvement in the future.

## Author’s Contributions

MSA, AWH, and MWHH conceived the idea and designed the main frame of this manuscript as part of MSA’s research work under the supervision of AWH. MSA processed and evaluated the post-thawed semen. MA and LA analyzed the data and statistical analysis. IDP critically read and revised the manuscript for intellectual content. All authors read and approved the final manuscript.
